# Novel, active, and uncultured hydrocarbon-degrading microbes in the ocean

**DOI:** 10.1128/aem.01224-24

**Published:** 2024-08-23

**Authors:** Kathryn L. Howe, Julian Zaugg, Olivia U. Mason

**Affiliations:** 1 Department of Earth, Ocean and Atmospheric Science, Florida State University, Tallahassee, Florida, USA; 2 Australian Centre for Ecogenomics, School of Chemistry and Molecular Biosciences, University of Queensland, St. Lucia, Queensland, Australia; University of Delaware, Lewes, Delaware, USA

**Keywords:** metagenome, metatranscriptome, MAG, hydrocarbon degradation, methane oxidation, oil degradation, gas degradation, genome assembly, read mapping

## Abstract

Given the vast quantity of oil and gas input to the marine environment annually, hydrocarbon degradation by marine microorganisms is an essential ecosystem service. Linkages between taxonomy and hydrocarbon degradation capabilities are largely based on cultivation studies, leaving a knowledge gap regarding the intrinsic ability of uncultured marine microbes to degrade hydrocarbons. To address this knowledge gap, metagenomic sequence data from the Deepwater Horizon (DWH) oil spill deep-sea plume was assembled to which metagenomic and metatranscriptomic reads were mapped. Assembly and binning produced new DWH metagenome-assembled genomes that were evaluated along with their close relatives, all of which are from the marine environment (38 total). These analyses revealed globally distributed hydrocarbon-degrading microbes with clade-specific substrate degradation potentials that have not been reported previously. For example, methane oxidation capabilities were identified in all *Cycloclasticus*. Furthermore, all *Bermanella* encoded and expressed genes for non-gaseous *n*-alkane degradation; however, DWH *Bermanella* encoded alkane hydroxylase, not alkane 1-monooxygenase. All but one previously unrecognized DWH plume member in the SAR324 and UBA11654 have the capacity for aromatic hydrocarbon degradation. In contrast, *Colwellia* were diverse in the hydrocarbon substrates they could degrade. All clades encoded nutrient acquisition strategies and response to cold temperatures, while sensory and acquisition capabilities were clade specific. These novel insights regarding hydrocarbon degradation by uncultured planktonic microbes provides missing data, allowing for better prediction of the fate of oil and gas when hydrocarbons are input to the ocean, leading to a greater understanding of the ecological consequences to the marine environment.

**IMPORTANCE:**

Microbial degradation of hydrocarbons is a critically important process promoting ecosystem health, yet much of what is known about this process is based on physiological experiments with a few hydrocarbon substrates and cultured microbes. Thus, the ability to degrade the diversity of hydrocarbons that comprise oil and gas by microbes in the environment, particularly in the ocean, is not well characterized. Therefore, this study aimed to utilize non-cultivation-based ‘omics data to explore novel genomes of uncultured marine microbes involved in degradation of oil and gas. Analyses of newly assembled metagenomic data and previously existing genomes from other marine data sets, with metagenomic and metatranscriptomic read recruitment, revealed globally distributed hydrocarbon-degrading marine microbes with clade-specific substrate degradation potentials that have not been previously reported. This new understanding of oil and gas degradation by uncultured marine microbes suggested that the global ocean harbors a diversity of hydrocarbon-degrading bacteria, which can act as primary agents regulating ecosystem health.

## INTRODUCTION

Annually, hundreds of millions of liters of oil are introduced to the ocean (1), raising global concerns regarding the adverse effects of oil pollution on biology and overall marine ecosystem function (2). This oil originates from both natural and anthropogenic sources and is sufficient to cover the world ocean in a thin layer of oil (1). The ocean is also a natural source of gaseous hydrocarbons such as methane, a potent greenhouse gas. It is estimated that the ocean contributes 1%–3% of global atmospheric methane (3). Thus, the consumption of oil and gas by microbes in the water column is an important ecosystem service, both in terms of marine environmental health and for the capture of greenhouse gases that would otherwise efflux to the atmosphere.

One such example of both the magnitude of oil and gas that can be input to the marine environment, and the important role that microbes play in consuming hydrocarbons, is the Deepwater Horizon (DWH) oil spill. From April to July 2010, nearly 5.3 × 10^11^ g of oil and 1.7 × 10^11^ g of natural gas were input to the Gulf of Mexico (GOM) water column (4). The depth at which the spill occurred (1,500 mbsl) prevented any photo-oxidation or oil recovery from the deep-sea water column. Therefore, microbial biodegradation of hydrocarbons was the primary mitigation taking place during the DWH spill in plume (5). Thus, the role of indigenous deep-sea microbes in oil and gas consumption is paramount in terms of bioremediation and the subsequent environmental impact of deep-sea oil spills, such as the DWH spill in 2010, and marine oil spills in general.

Analysis of uncontaminated deep-sea samples revealed a diversity of microbes in the deep-sea water column in the GOM (6, 7), the majority of which are uncultured and, therefore, have unknown physiologies. This diversity decreased in seawater contaminated with DWH hydrocarbons, relative to uncontaminated samples (6, 8). The shifts in the taxonomic composition and abundance of indigenous deep-sea microbiota that led to decreasing diversity is well documented (6, 7, 9–13). These studies revealed a pattern in succession with *Oceanospirillales* (now recognized to be *Bermanella* and will be referred to as such in the results of this study) dominating early in the spill followed by *Colwellia*, *Cycloclasticus,* and finally by methylotrophs. The changes in the dominant microbial players were attributed to variation in the supply of different hydrocarbons during the spill (12). For example, *Oceanospirillales* dominated during the period of unrestricted release of oil, when the concentration of *n*-alkanes and cycloalkanes was highest (12). In June 2010, partial capture of DWH hydrocarbons commenced, leading to the dominance of benzene, toluene, ethylbenzene, and xylene (BTEX) and natural gases in the plume (4), which coincided with an increase in the abundance of *Colwellia* and *Cycloclasticus* (12).

Although more than a decade has passed since the DWH spill, an information gap regarding microbial hydrocarbon degradation persists, which diminishes our ability to understand the ecosystem effects of both past and future oil spills in the marine environment. For example, although gaseous hydrocarbons were the most abundant hydrocarbon input during the spill (4), and evidence that oxidation of methane [and ethane and propane (10)] was an active microbial process (11, 14), methane oxidation capabilities remain largely unattributed to any specific microbial clade (5). A variety of approaches including particulate methane monooxygenase (*pmo*) gene phylogenetic reconstruction (14), stable isotope probing (SIP) coupled with 16S rRNA gene sequencing (7), and metagenome (6) and metatranscriptome sequence annotation (6, 8) revealed a diversity of *pmo* genes and transcripts, but taxonomy and function of indigenous methane-oxidizing bacteria was not definitively linked through genome assembly.

Non-gaseous straight chain *n*-alkanes, such as *n*-pentane and *n*-hexane, the cyclic alkanes cyclopentane and cyclohexane, and BTEX aromatics were also abundant in the DWH deep-sea plume (15, 16). Polycyclic aromatic hydrocarbons (PAHs) were observed in the plume at levels that would have acute toxicity effects (17), at the surface (9), and on the seafloor (18). The degree of resolution on microbial consumption of non-gaseous hydrocarbons varies. For example, single amplified genome (SAG) recovery revealed *n*-alkane and cycloalkane degradation capabilities in the plume which was ascribed to indigenous *Oceanospirillales* (6). This *Oceanospirillales* SAG has genes coding for alkane monooxygenase (*alkB*), cyclohexanol dehydrogenase, and cyclohexanone monooxygenase (6). Although the *Oceanospirillales* SAG was derived from the same plume samples in Mason et al. (6), that were further analyzed in this current study, the 16S rRNA gene encoded in the SAG was 95% similar to the dominant, indigenous *Oceanospirillales* and the SAG recruited a low number of complementary metagenomic and metatranscriptomic reads. Therefore, how well it represented the hydrocarbon degradation pathways in the dominant *Oceanospirillales* in the plume is not clear.

Even less is known about what plume microbes were capable of degrading aromatics. Culturing experiments have revealed that *Cycloclasticus* can grow on aromatic compounds (1), including toluene (19, 20), xylene (20) and PAHs (21, 22). SIP experiments revealed a possible role for *Colwellia* in degrading benzene (7). Yet, information on specifically which indigenous deep-sea microbes are capable of degrading aromatic compounds remains a persistent knowledge gap.

Thus, much remains unknown regarding the degradation capabilities of uncultured, indigenous deep-sea microbes more than a decade since the DWH oil spill, during which time new and improved metagenome assembly and classification tools have become available. We have taken advantage of such tools to assemble the metagenomic data presented in Mason et al. (6), obtaining 12 DWH metagenome-assembled genomes (MAGs) of medium to high quality (23). Previous attempts to assemble these data were largely unsuccessful (6), thus important information regarding degradation potential and identity remain unresolved. To expand our understanding of hydrocarbon degradation, nutrient acquisition, chemotaxis, and motility, beyond the GOM to the global ocean, these DWH MAGs were analyzed together with 26 close relatives, that were determined as such by assessing phylogenetic relationships using bacterial single-copy marker proteins. The results of this analysis provided missing linkages between taxonomy coupled with function, the lack of which previously hampered our understanding of microbial hydrocarbon oxidation in the marine environment. This new information allows for more accurate prediction of the ecological consequences of hydrocarbons, in all of its forms, that are input to the ocean.

## MATERIALS AND METHODS

### Sample collection

As described in Mason et al. (6), seawater was filtered through 0.2 µm diameter filters from stations sampled in the Gulf of Mexico during two cruises from 27 May to 2 June 2010 on the R/V Ocean Veritas and R/V Brooks McCall. All samples were collected at plume depth (~1,100 mbsl). These samples were OV011 a near-plume, proximal station that was located 1.8 km from the wellhead, BM58 a far-plume, distal station, 10.8 km from the wellhead, and OV003 an uncontaminated station 37 km from the wellhead. Further details regarding sample collection were provided in Mason et al. (6) and Hazen et al. (9).

### DNA and RNA extraction, processing, and sequencing

A detailed extraction protocol was provided in Mason et al. (6). Briefly, DNA was extracted from microbial cells collected onto filters using a modified Miller method (24), with the addition of a pressure lysis step to increase cell lysis efficiency. Sample material was transferred to a Lysing Matrix E tube (MP Biomedicals, Solon, OH) and the samples were subjected to bead beating, followed by centrifugation at which time chloroform was added to the supernatant. After centrifugation, the aqueous phase clean-up procedures followed the instructions in the MoBio Soil DNA extraction kit.

Total RNA was processed as described in Mason et al. (6). In summary, total RNA from the proximal and distal plume stations was amplified, followed by first-strand synthesis of cDNA and double-stranded cDNA. Poly(A) tails were removed from purified cDNA by digesting purified DNA with *Bpm*I for 3 h at 37°C. Digested cDNA was subsequently purified.

To increase yields required for sequencing, DNA and cDNA were amplified by emulsion PCR. A detailed description of this method can be found in reference (25). Amplified DNA was sequenced using the Illumina GAIIx with 2 × 114 bp pair-end technology, resulting in 14–17 Gb of sequence read data per sample. These reads are publicly available in NCBI (PRJNA336903-05), in IMG Gold Study ID Gs0063184 and on the Mason server http://mason.eoas.fsu.edu in the DWH_plume directory. Amplified cDNA was sequenced using the Illumina GAIIx sequencing platform with 2 × 100bp pair-end technology. These reads are publicly available in NCBI (PRJNA839076) on on the Mason server in DWH_plume. MAGs assembled and analyzed herein are available in NCBI under sample BM58 (PRJNA336904) MAG accession numbers JAVTJO000000000- JAVTJR000000000, sample OV011 (PRJNA336903) MAG accession numbers JAVTJS000000000-JAVTJW000000000, sample OV003 (PRJNA336905) MAG accession numbers JAVTJX000000000-JAVTJZ000000000 and on the Mason server in DWH_plume.

### Metagenome assembly and annotation and metagenomic and metatranscriptomic read mapping

The quality of paired-end metagenomic reads was assessed, and adapter sequences identified, using FastQC (v.0.10.1, http://www.bioinformatics.babraham.ac.uk/projects/fastqc/). Low-quality reads and adapters were subsequently removed using Trimmomatic (v.0.36, ILLUMINACLIP:TrueSeq and over represented adapters:2:30:10, LEADING:3, TRAILING:3, SLIDINGWINDOW:4:15, MINLEN:50) (26). Those reads lacking a mate pair were removed. Reads were assembled using MetaSPAdes (v.3.13) (27) with default parameters. Contigs less than 500 bp were removed using BBMap (v.38.35, https://sourceforge.net/projects/bbmap/). Filtered reads were mapped onto their respective scaffolds using CoverM “make” (v.0.2.0, https://github.com/wwood/CoverM), with CoverM “filter” used to remove low-quality mappings (minimum identity 95% and minimum aligned length of 50 bp). Scaffolds for each sample were binned using UniteM (v.0.0.15, https://github.com/dparks1134/UniteM), with a minimum contig length of 1,500 bp, and Maxbin (v.2.2.4) (28), MetaBAT (v.0.32.5) (29), and MetaBAT2 (v.2.12.1) (30) binning methods (max40, max107, mb2, mb_verysensitive, mb_sensitive, mb_specific, mb_veryspecific, and mb_superspecific). From this assembly approach, 12 MAGs were obtained (Table 1), hereinafter referred to as DWH MAGs. The completeness and contamination of all bins were calculated by CheckM (v.1.0.12) (31). These MAGs were medium (>50% complete and <10% contamination) and high quality (>90% complete and <5% contamination), except the three *Colwellia*, which met one of the two criteria (Table 1). Taxonomy was assigned using the Genome Taxonomy Toolkit (GTDB-Tk, v.2.3.0; with reference to GTDB R08-RS214) (32) with up to 120 bacterial single-copy marker proteins, which are predominantly ribosomal [see Table S6 from Parks et al. (33)]. The GTDB-Tk phylogenetic tree based on the alignment of concatenated single-copy marker proteins was viewed using Archaeopteryx (34) to identify close relatives of the MAGs using phylogeny with monophyly and branch length as decision-making criteria for inclusion (see Fig. S1). These 26 additional genomes were from marine microbes and were included in all subsequent analyses to extend the data set to include microbes that were sampled from the global ocean (Table 1). These genomes are referred to as non-DWH to distinguish those assembled from the Mason et al (6) data set (DWH MAGs). Details regarding non-DWH genomes, including genome completeness, contamination, and sampling location are included in Table 1.

**TABLE 1:** Detailed information regarding genome statistics, including completeness and contamination percentages, sampling locations, and depths

Genome	Genome size	Contig count	N50 contigs	% Completeness	% Contamination	% GC	Sample site	Sample depth (mbsl)	Reference
**BM58_1_Bermanella ^*a*^**	2,983,408	527	8,317	92.35	0.85	52.04	DWH distal plume	1,179	This paper
**OV003_3_Bermanella**	2,094,764	704	3,220	77.79	1.73	52.05	DWH uncontaminated, plume depth	1,020	This paper
**OV011_4_Bermenalla**	2,533,940	328	10,666	89.38	0.10	52.57	DWH proximal plume	1,207	This paper
GCA_000153565.1_ASM15356v1 _Bermanella_marisrubri_RED65	3,552,236	47	122,078	98.91	0.89	43.96	Red Sea	1	Pinhassi et al. (35)
GCA_002162985.1_ASM216298v1 _Bermanella_sp._47_1433_sub80_T6	2,540,546	393	9,226	80.20	3.06	47.43	DWH microcosms	1,500	Hu et al. (36)
Bermanella_DWH_O._Desum_v2	1,622,697	1,012	1,608	58.3		52.72	DWH distal plume	1,179	doi:10.6084 /m9.figshare.5633344
GCA_002683575.1_ASM268357v1 _Bermanella_sp._CPC64	3,164,348	158	27,520	91.95	0.55	44.29	North Atlantic Ocean	5	Tully et al. (37)
**BM58_2_Cycloclasticus**	3,525,309	334	24,676	94.82	1.88	44.11	DWH distal plume	1,179	This paper
**OV011_1_Cycloclasticus**	2,388,444	324	9,911	87.77	1.22	45.72	DWH proximal plume	1,207	This paper
GCA_002162695.1_ASM216269v1 _46_120_T64_Cycloclasticus	2,210,308	17	200,835	97.59	0.36	45.91	DWH microcosms	1,500	Hu et al. (36)
GCA_002101205.1_ASM210120v1 _Cycloclasticus_sp._symbiont_of_Poecilosclerida_sp.	1,916,434	78	99,813	91.27	1.72	42.74	GOM deep-sea asphalt seep	3,000	Rubin-Blum et al. (38)
GCA_002101235.1_ASM210123v1_Cycloclasticus_sp. _symbiont_of_Bathymodiolus_heckerae_specimen_P	2,130,426	123	82,558	97.13	2.07	42.21	GOM deep-sea asphalt seep	3,000	Rubin-Blum et al. (38)
GCA_002733135.1_ASM273313v1 _Cycloclasticus_sp._NORP92	1,756,937	24	118,716	84.54	0.01	42.22	Mid-Atlantic Ridge crustal fluid	4,300	Tully et al. (39)
GCA_002292375.1_ASM229237v1 _Methylococcaceae_bacterium_UBA975	2,328,365	28	137,214	97.04	2.41	44.76	North Sea seawater	700	Parks et al. (33)
**BM58_3_Colwellia**	3,937,173	1,316	3,073	52.88	11.90	36.09	DWH distal plume	1,179	This paper
**BM58_4_Colwellia**	4,289,381	935	5,460	60.37	13.51	39.68	DWH distal plume	1,179	This paper
**OV011_6_Colwellia**	2,643,374	1,008	2,536	41.45	3.08	39.43	DWH proximal plume	1,207	This paper
GCA_002162675.1_ASM216267v1 _Colwellia_sp._39_35_sub15_T18	4,011,205	74	107,820	94.94	1.38	38.62	DWH microcosms	1,500	Hu et al. (36)
GCA_002163005.1_ASM216300v1 _Colwellia_psychrerythraea_38_32_sub10_T18	4,614,230	128	63,544	91.25	3.41	38.11	DWH microcosms	1,500	Hu et al. (36)
GCA_000012325.1_ASM1232v1 _Colwellia_psychrerythraea_34H	5,373,180	1	5,373,180	100	1.68	38.01	Arctic sea ice	305	Methé et al. (40)
GCA_002733765.1_ASM273376v1 _Colwellia_sp._NORP29	4,048,040	41	181,020	96.66	1.90	37.00	Mid-Atlantic Ridge crustal fluid	4,300	Tully et al. (39)
GCA_000764225.1_ASM76422v1 _Colwellia_psychrerythraea_ND2E	5,154,850	57	297,116	100.00	2.38	38.08	Eastern Mediterranean Sea	495	Techtmann et al. (41)
GCA_003521815.1_ASM352181v1 _Colwellia_sp._UBA8680	2,348,646	296	16,886	87.56	0.26	37.30	Coastal seawater	NA	Parks et al. (33)
**OV003_4_UBA11654**	2,105,006	577	4,225	67.6	10.30	39.82	DWH uncontaminated, plume depth	1,020	This paper
**OV011_5_UBA11654**	2,016,421	591	3,744	66.05	14.40	40.25	DWH proximal plume	1,207	This paper
GCA_001629325.1_ASM162932v1 _Gammaproteobacteria_bacterium_REDSEA-S03_B5	1,166,919	135	12,285	67.34	1.22	38.75	Red Sea	47	Haroon et al. (42)
GCA_003449795.1_ASM344979v1 _Gammaproteobacteria_bacterium_UBA11654	1,179,807	222	7,290	57.36	0.00	38.34	Marine water column	NA	Parks et al. (33)
GCA_002170535.1_ASM217053v1 _Methylococcaceae_bacterium_TMED69	1,973,610	10	233,566	82.22	1.83	38.55	Mediterranean Sea	72	Tully et al. (37)
GCA_002691305.1_ASM269130v1 _Gammaproteobacteria_bacterium_MED656	1,477,774	70	25,209	65.68	1.52	39.33	Mediterranean Sea	500	Tully et al. (37)
GCA_002691465.1_ASM269146v1 _Gammaproteobacteria_bacterium_MED641	1,402,833	41	49,343	88.45	1.68	38.62	Mediterranean Sea	500	Tully et al. (37)
GCA_002717445.1_ASM271744v1 _Gammaproteobacteria_bacterium_SP335	1,506,851	31	59,694	80.99	3.05	39.84	South Pacific Ocean	500	Tully et al. (37)
GCA_002718375.1_ASM271837v1 _Gammaproteobacteria_bacterium_SP325	878,692	24	48,826	59.48	1.72	38.34	South Pacific Ocean	500	Tully et al. (37)
**OV011_2_SAR324**	2,447,870	421	7,630	87.38	0.00	43.02	DWH proximal plume	1,207	This paper
**OV003_1_SAR324**	2,403,622	419	8,188	88.32	2.15	42.36	DWH uncontaminated, plume depth	1,020	This paper
GCA_000213335.2_ASM21333v2 _SAR324_cluster_bacterium_SCGC_AAA001-C10	2,158,589	441	22,585	51.53	0.00	41.11	South Atlantic	800	Swan et al. (43)
GCA_001627865.1_ASM162786v1 _SAR324_cluster_bacterium_REDSEA-S27_B3	3,302,781	82	71,211	90.94	0.00	42.85	Red Sea	500	Haroon et al. (42)
GCA_002082305.1_ASM208230v1 _SAR324_cluster_bacterium_SAR324-CTD7B	2,804,129	148	26,077	89.58	0.00	42.30	South Mid-Atlantic Ridge	2,750	Cao et al. (44)
GCA_002716165.1_ASM271616v1 _SAR324_Deltaproteobacteria_bacterium_SP68	1,345,594	58	22,845	59.27	0.84	35.99	South Pacific Ocean	500	Tully et al. (37)

^*a*^ Genomes shown in bold represent new MAGs that were assembled in this study. GenBank accession links are embedded in these MAG names.

All genomes were analyzed using Anvi’o (v.6.2) (45). In Anvi’o, Prodigal was used to identify open reading frames (ORFs). ORFs were annotated with “anvi-run-pfams” and “anvi-run-ncbi-cogs,” based on the Pfam (46) and NCBI’s Clusters of Orthologous Genes (COG) (47) databases, respectively. In addition to Pfam and COG annotations, ORFs were compared to NCBI’s non-redundant RefSeq protein data set (accessed on 03/10/2020) (48) using blastx with DIAMOND (v.0.9.30) (49) and CANT-HYD, the curated database for hydrocarbon degradation genes (50), with an e-value cutoff of 10^−4^ for both. Functional annotations were confirmed when three of four databases (COGs, Pfams, RefSeq, and CANT-HYD) agreed. However, the CANT-HYD database does not include archetype sequences for ferredoxin, catechol dioxygenase, cylcohexanone monooxygenase, or flavin-containing monooxygenases. In this case, functional annotations were confirmed when two of the remaining three databases, Pfams, COGs, and RefSeq, had the same annotation.

To determine genome, gene and transcript abundances, metagenomic (MG) and metatranscriptomic (MT) reads were mapped to DWH MAGs and non-DWH genomes using Bowtie 2 (v.2.3.4.1) (51) following the script provided at https://merenlab.org/data/tara-oceans-mags/ and used in Thrash et al. (52) and more recently as the RRAP pipeline (53). Before mapping cDNA reads, ribosomal RNA was subtracted using riboPicker (v.0.4.3) with the default settings (54). Low-quality read mappings were filtered using BamM (v.1.7.3, http://ecogenomics.github.io/BamM/) with --percentage_id 0.95 --percentage_aln 0.75. Alignment summary statistics were obtained using SAMtools (v.1.7-2) (55) command idxstats with default settings. Subsequently, genome, gene, and transcript abundances were normalized by calculating reads per kilobase per million (RPKM) mapped reads according to Mortazavi et al. (56), and detailed in Thrash et al. (52) and Kojima et al. (53). Following normalization, average RPKM values were calculated, with low values being below average and high values being above average.

### Pangenomic analysis

A comparative genomic analysis of the 12 DWH MAGs and 26 non-DWH genomes was performed using the Anvi’o pangenomic workflow v.2 (45). Hits between gene clusters, the representative sequences of predicted ORFs that are grouped together based on homology at the translated DNA sequence level, and bacterial single-copy core genes were identified using HMMER and hidden Markov models (anvi-run-hmms). The pangenome was generated using DIAMOND (49) as part of the “anvi-pan-genome” program, which was run in sensitive mode with hierarchical clustering enforced. Core genes encoded in all 38 genomes were identified, as was a DWH pangenome for the core genes among only the 12 DWH MAGs. Average nucleotide identity (ANI) among all genomes was calculated using Sourmash with --containment --ani --ksize 31 (57).

## RESULTS

### DWH MAG assembly, taxonomy, and close relatives from the global ocean

Twelve MAGs were assembled from the DWH proximal plume (OV011), distal plume (BM58), and a non-oil contaminated sample collected at plume depth (OV003) from metagenomes that were presented in Mason et al. (6) but were not assembled and binned. These MAGs represent four different clades in the Gammaproteobacteria, as well as the SAR324 clade [formerly part of the Deltaproteobacteria, now a candidate phylum (58)]. The new DWH MAGs presented in the current study are classified as *Bermanella* (3), *Colwellia* (3), *Cycloclasticus*, (2), UBA11654, and SAR324 (two each) (Table 1). These MAGs averaged 75% completeness and had low contamination (avg. 5%) (Table 1).

While most similar to one another, DWH MAGs were also closely related to non-DWH genomes as identified from phylogenetic analysis (Fig. S1). These non-DWH genomes were medium to high quality with an average of 83% completeness and 1.36% contamination (Table 1). These microbes were sampled from the marine surface to the deep-sea water column, primarily from sites that were free from hydrocarbon contamination (Table 1). For example, as shown in Table 1, these close relatives were sampled in the Red Sea (35, 42), the North Atlantic Ocean (37), South Pacific Ocean (37), Mediterranean Sea (41, 59), the North Sea (33), and also from crustal fluids collected on the flanks of the Mid-Atlantic Ridge (39, 44), symbionts of mussels and sponges living at hydrocarbon seeps (38), and microcosms with Macondo oil (36).

**Fig 1:**
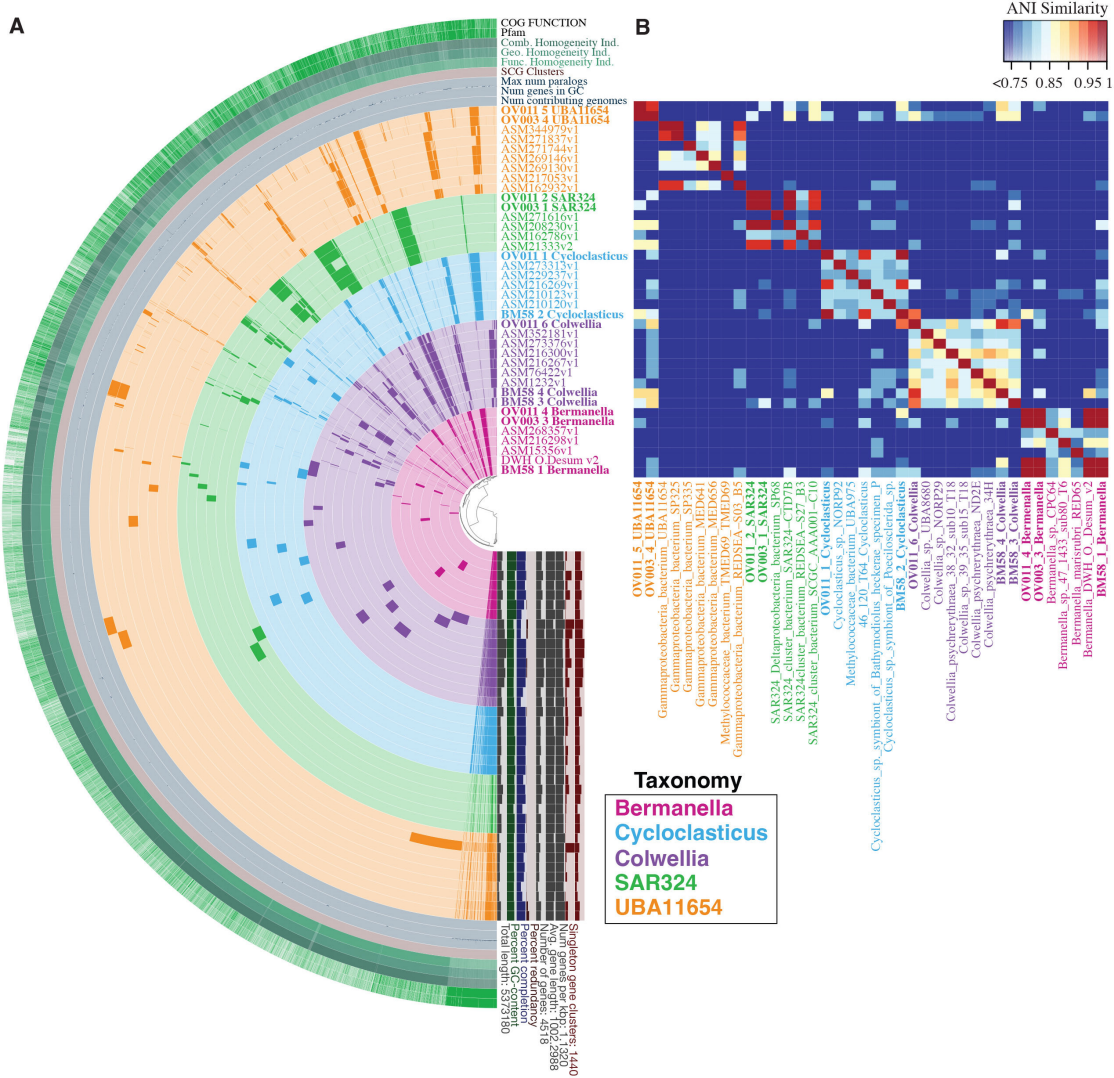
Phylogram (**A**) and ANI plot (**B**) of all 38 MAGs and genomes analyzed. ANI plot (**B**) only shows ANI above 75%. Genome names are color coded by taxonomy with DWH MAGs in bold text.

Similar to the results of the phylogenetic analysis, ANI calculations showed DWH MAGs exhibited the most similarity with other DWH MAGs within the same clades (Fig. 1). However, there were several other genomes that also had high ANI with DWH MAGs. Most notably, the “DWH O. Desum v2” MAG (60), which was previously assembled from the metagenomic data presented in Mason et al. (6), had >99% ANI with DWH *Bermanella* MAGs (Fig. 1). DWH *Bermanella*, *Cycloclasticus*, and *Colwellia* MAGs (Fig. 1) were 70% to more than 90% similar to MAGs described in Hu et al. (36) that were obtained from microcosms with Macondo oil, although DWH *Colwellia* MAGs were equally similar to *Colwellia psychrerythraea* 34H, isolated from Arctic sea ice (40). DWH UBA11654 MAGs and DWH SAR324 were most similar to each other at 98%, with 75% or less in similarity with non-DWH UBA11654 genomes (Fig. 1). DWH SAR324 MAGs were most similar to each other and two non-DWH SAR324 MAGs (Fig. 1).

### Microbial abundances and transcriptional activity

**Fig 2:**
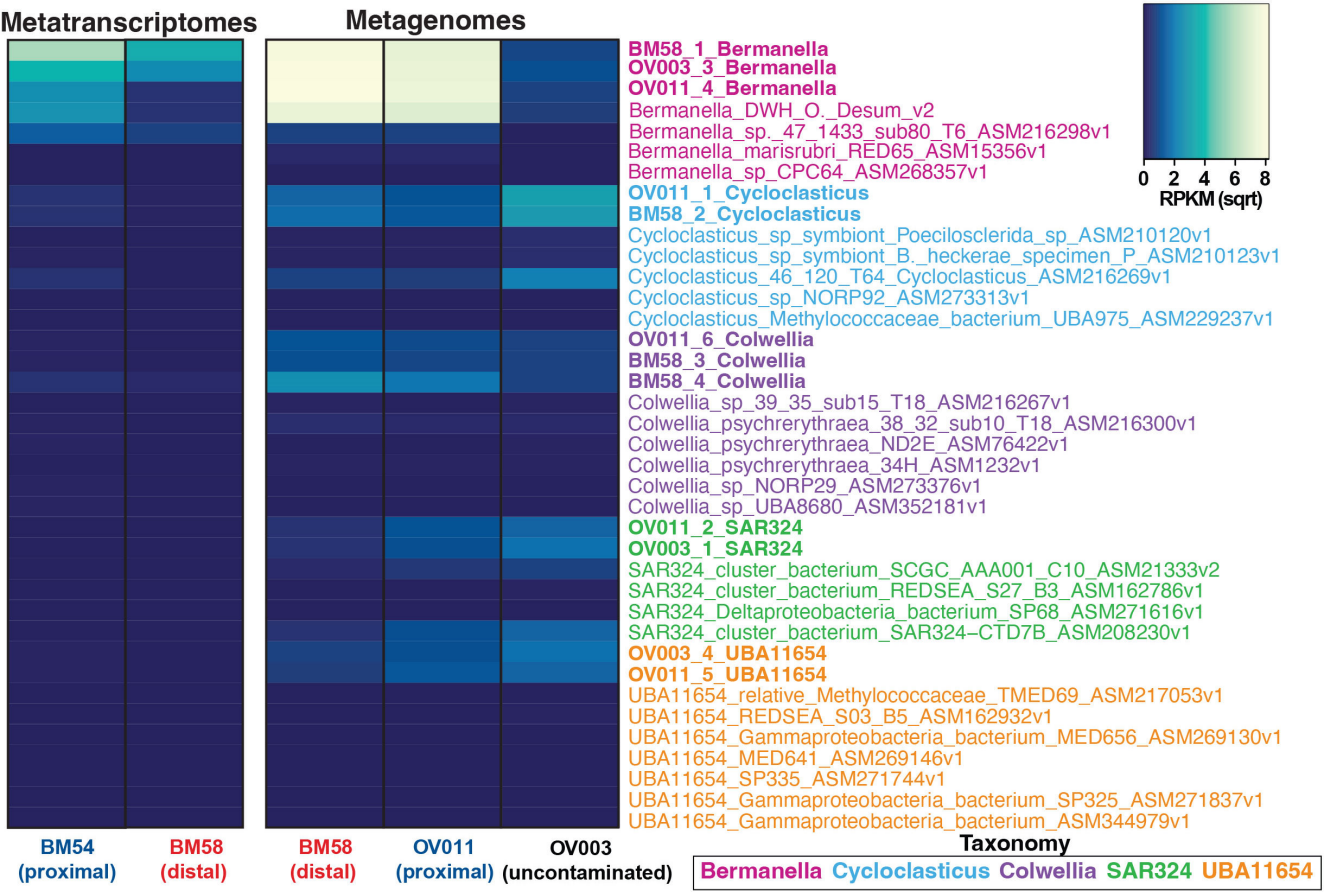
Heatmap of microbial abundance and transcriptional activity as determined by read recruitment of metagenomic and metatranscriptomic data to the 38 MAGs and genomes analyzed in this study. Genome names are color coded by taxonomy with DWH MAGs in bold text.

Microbial abundances and transcriptional activity were determined by mapping unassembled metagenomic and metatranscriptomic reads from Mason et al. (6) to the 38 DWH and non-DWH genomes included in this study. Read counts were normalized by determining RPKM and are reported as low if RPKM values were below average, or high if RPKM values were above average. Of the 38 genomes analyzed, DWH *Bermanella* had the highest abundances (Fig. 2). Specifically, DWH *Bermanella* MAGs had MG RPKM values of 67 in the distal plume and 55 in the proximal plume and MT values of 32 in the proximal plume and 12 in the distal plume (Fig. 2). DWH O. Desum v2 was also abundant (MG RPKM ranged from 47 to 53), but was not highly represented when evaluating gene expression (Fig. 2). Non-DWH *Bermanella*, while less abundant, did recruit MG and MT reads (Fig. 2).

DWH *Cycloclasticus* and *Colwellia* MAGs were abundant, but less so than *Bermanella* with maximum MG RPKM values of 9 (Fig. 2). These MAGs recruited MT reads, but RPKM values were low in comparison to *Bermanella* (Fig. 2). Non-DWH *Cycloclasticus* and *Colwellia* and DWH and non-DWH UBA11654 and SAR324 were not abundant with below average MG and MT read recruitment (Fig. 2).

### Hydrocarbon degradation

#### Gaseous hydrocarbons

**Fig 3:**
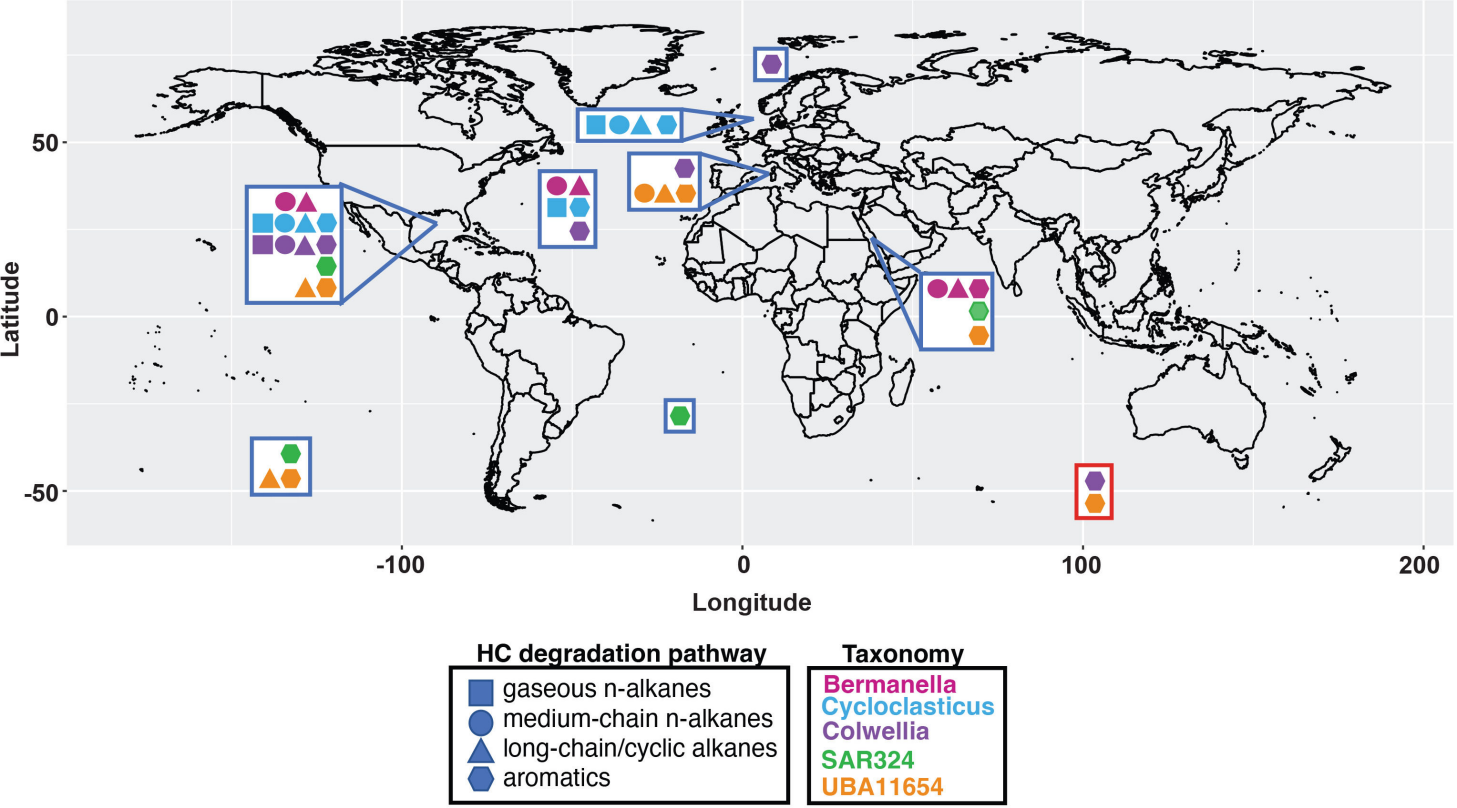
Global distribution of sample locations of genomes analyzed, with their hydrocarbon degradation capabilities shown by shape and color coded by taxonomy. The red box encloses genomes that were assembled from the marine environment, but the exact location was not provided in Parks et al. (33). The map was created using R with ggplot2.

**Fig 4:**
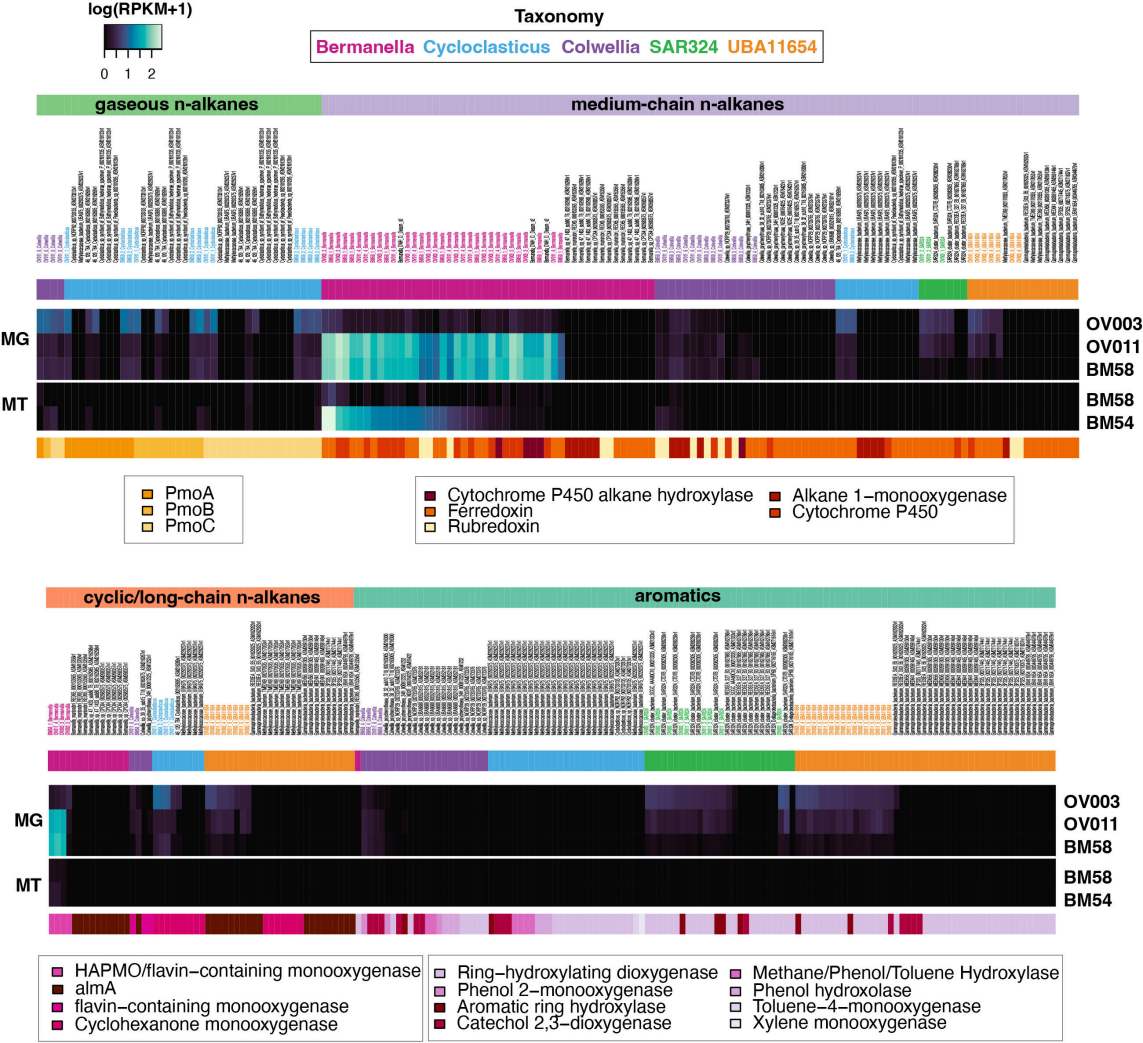
Heatmap showing the abundance of genes and transcripts that code for hydrocarbon degradation based on read recruitment of metagenomic (MG) and metatranscriptomic (MT) sequence data. DWH MAG genes are shown in bold text and color coded by taxonomy. Genes encoded in non-DWH genomes are shown in black text.

All *Cycloclasticus* genomes, including DWH *Cycloclasticus* MAGs, coded for methane oxidation (Fig. 3 and 4). Outside of the *Cycloclasticus,* only a DWH *Colwellia* MAG encoded methane oxidation (Fig. 3 and 4). Specifically, all *Cycloclasticus* and one *Colwellia* encoded methane/ammonia monooxygenase, subunits A–C (Fig. 4). Only these DWH MAGs and one Macondo oil microcosm MAG harbored *pmoABC* genes that recruited MG and MT reads (Fig. 4). From the uncontaminated deep-sea sample, DWH *Cycloclasticus* and *Colwellia pmoABC* genes were the most abundant, reflected in their recruitment of the greatest number of reads of any genes involved in hydrocarbon degradation analyzed herein (MG RPKM maximum was 15; Fig. 4). Specifically, DWH *Cycloclasticus pmoABC* had the highest MG and MT RPKM values in proximal and distal plumes followed by DWH *Colwellia* (Fig. 4).

All genomes that recruited reads to *pmoABC* genes also encoded additional steps culminating in a partial methane oxidation pathway, including a short-chain alcohol dehydrogenase and generic aldehyde dehydrogenase (Fig. S2). Additionally, multiple formaldehyde oxidation pathways were encoded, with the tetrahydrofolate pathway having the highest MG recruitment (Fig. S2), while no MT reads were recruited (Fig. S2). Of the remaining genomes that encoded *pmoABC,* but lacked read recruitment (four non-DWH *Cycloclasticus*), two encoded a partial pathway while the other two encoded a full pathway including formate dehydrogenase (Fig. S2). However, only *Cycloclasticus* sp. symbiont of *Bathymodiolus heckerae* specimen P glutathione-dependent (GSH) pathway genes recruited MG reads, but these genes were not expressed (Fig. S2).

#### Non-gaseous *n*-alkanes

All *Bermanella* encoded enzymes for medium-chain (C_5_–C_26_) *n*-alkane degradation, but there was more than one pathway observed. For example, all DWH *Bermanella* MAGs and DWH O. Desum v2 coded for genes in the cytochrome P450 family, not alkane 1-monooxygenase (AlkB), an enzyme used by *Alcanivorax borkumensis* in the degradation of medium-chain *n*-alkanes. Specifically, a cytochrome P450 alkane hydroxylase (CYP153) was encoded in all DWH *Bermanella* MAGs and DWH O. Desum v2. These genes were highly abundant in the plume, with MG RPKM values up to 54 (Fig. 4). While present in the uncontaminated sample, RPKM values were low (below average) relative to the plume samples (Fig. 4). All DWH *Bermanella* MAGs CYP153 genes were expressed in the plume samples, as was DWH O. Desum v2 (Fig. 4). A DWH *Colwellia* MAG also encoded a CYP153 gene, with low abundances in the distal and proximal plumes, but was undetectable in the uncontaminated sample, and no gene expression was observed (Fig. 4).

Three DWH *Bermanella* MAGs and DWH O. Desum v2 that encoded CYP153 also encoded a generic cytochrome P450 (not annotated as alkane hydroxylase) and ferredoxin. These genes were abundant with above-average MG and MT read recruitment (Fig. 4). Three of these *Bermanella* also encoded and expressed ferredoxin reductase genes (Fig. 4), suggesting a three-domain CYP153 architecture (61) for non-gaseous *n*-alkane degradation.

In contrast to DWH *Bermanella*, the *alkB* gene was present in the genomes of all non-DWH *Bermanella*, in two DWH *Colwellia* MAGs, and non-DWH *Cycloclasticus* and UBA11654 (Fig. 4). These *alkB* genes recruited MG reads from the proximal plume, distal plume and the uncontaminated sample, while the *alkB* gene encoded by *Bermanella* sp. 47 1433 sub80 T6 (36) recruited a low level of reads and only from the distal plume (Fig. 4). The only actively expressed *alkB* genes were encoded by a DWH *Colwellia* MAG, which recruited MT reads from both plume samples (Fig. 4).

AlkB is part of the two-domain architecture (61) with rubredoxin mediating the second step in alkane oxidation (62). Although no DWH *Bermanella* MAGs harbor *alkB*, all had rubredoxin genes, as did non-DWH *Bermanella marisrubri* RED65 and *Bermanella* sp. CPC64, while DWH O. Desum v2 and *Bermanella* sp. 47 1433 sub80 T6 lacked this gene. DWH *Bermanella* rubredoxin genes were abundant in the proximal and distal plume (RPKM maximum was 14), but less so in the uncontaminated sample (Fig. 4). These genes were expressed in both plume samples with RPKM values up to 14 (Fig. 4). The remaining rubredoxin genes were not abundant, recruiting few to no MG and MT reads (Fig. 4).

#### Long-chain *n*-alkanes and cyclic alkanes

There was a diversity of genes encoding enzymes for long-chain *n*-alkane (>C_18_) and cyclic alkane degradation in all clades analyzed here, except SAR324. In particular, this was a ubiquitous pathway in DWH and non-DWH *Bermanella*. For example, all DWH *Bermanella*, including DWH O. Desum v2, and two non-DWH *Bermanella* coded for genes that were most similar (58%) to a 4-hydroxyacetophenone monooxygenase (HAPMO) gene in a MAG from a subseafloor aquifer (39). These proteins were equally as similar to non-HAPMO enzymes annotated as flavin-containing monooxygenases. We also would note that these proteins have low-sequence similarity with AlmA and with *A. borkumensis’* cyclohexanone monooxygenase (30% and 24%, respectively). Overall, these flavin-containing monooxygenases were highly abundant with MG RPKM abundances up to 58 in plume samples, but were less abundant in the uncontaminated sample and expressed at low levels in the plume samples (Fig. 4). Of the long-chain and cyclic alkane gene annotations, those encoded by the aforementioned *Bermanella* were the only genes that were expressed (Fig. 4).

Other flavin-containing monooxygenases were identified in DWH and non-DWH *Bermanella, Cycloclasticus,* and UBA11654. These flavin-binding monooxygenase genes were most abundant in the uncontaminated sample, compared to the plume samples (Fig. 4). None of these genes were expressed (Fig. 4). In this group, DWH and non-DWH *Cycloclasticus* and UBA11654 and non-DWH *Bermanella* genes were annotated as *almA*. The AlmA enzyme is a well-known flavin-binding monooxygenase that functions as an alkane hydroxylase in *Alcanivorax hongdengensis* and *Alcanivorax dieselolei*, allowing for microbial degradation of long-chain *n*-alkanes (63, 64).

Finally, cyclohexanone monooxygenases, a Baeyer-Villiger monooxygenase that is involved in degradation of cyclic alkanes, was identified in DWH and non-DWH *Cycloclasticus* and non-DWH UBA11654. Only DWH *Cycloclasticus* cyclohexanone monooxygenases recruited reads, with an MG maximum RPKM of 10 in the uncontaminated sample and RPKM values of <1 in the plume sample (Fig. 4). None of these genes recruited MT reads (Fig. 4).

#### Aromatics

Members of all clades, including one non-DWH *Bermanella*, encoded for aromatic hydrocarbon degradation genes which were primarily ring-hydroxylating dioxygenases, with a lower number of catechol 2,3-dioxygenases. DWH and non-DWH members of *Colwellia*, UBA11654, and SAR324 were the only clades to recruit any reads to these genes, with the largest number of MG reads from the distal plume, although RPKM values were low compared to other hydrocarbon degradation pathways (Fig. 4). Despite the abundance of BTEX hydrocarbons in the plume (4), only two genes specifically coding for BTEX degradation, toluene-4-monooxygenase and xylene monooxygenase, were observed in any genome, and only in the non-DWH *Cycloclasticus Methylococcaceae bacterium* UBA975 (33), which did not recruit any MG or MT reads (Fig. 4). Overall, these genes were most abundant in the uncontaminated, plume depth station, with non-DWH and DWH SAR324 and UBA11654 genes recruiting the greatest number of reads (maximum MG RPKM was 6; Fig. 4). None of the aromatic hydrocarbon-degrading genes present in the genomes analyzed here were expressed (Fig. 4).

### Nitrogen, sulfur, phosphorus, and iron

**Fig 5:**
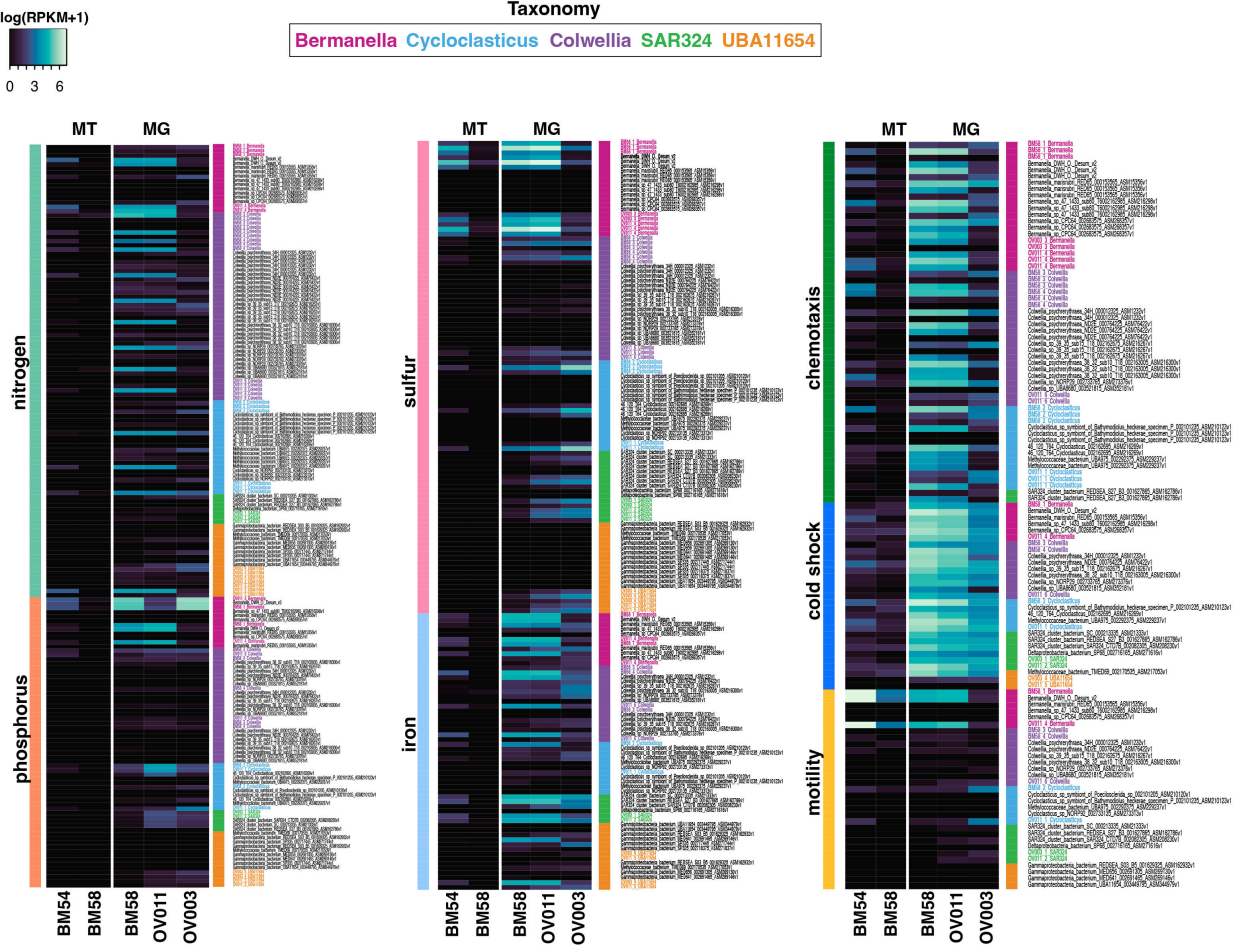
Heatmap showing the abundance of genes and transcripts coding for anaerobic respiration, nutrient acquisition, chemotaxis, motility, and cold shock based on read recruitment of metagenomic (MG) and metatranscriptomic (MT) sequence data. DWH MAG genes are in bold text and color coded by taxonomy. Non-DWH genes are in black text.

In evaluating the capacity for carrying out anaerobic respiration through denitrification, most *Colwellia* genomes in this study encoded the necessary enzymes to carry out complete denitrification (Fig. 5). Specifically, DWH and non-DWH *Colwellia* nitrate and nitrite reductase genes were abundant and expressed (Fig. 5). However, their nitric oxide reductase and nitrous oxide reductase were not abundant (Fig. 5). Outside of the *Colwellia,* DWH and non-DWH *Bermanella*, *Cycloclasticus*, and UBA11654 genomes encoded steps in the denitrification pathway, but none had the capacity for complete denitrification (Fig. 5). Beyond respiratory genes, all clades encoded nitrate/nitrite transporters, with one DWH *Colwellia* and DWH O. Desum v2 having the most abundant transporter genes, while DWH *Bermanella* and DWH UBA11654 nitrate/nitrite transporters were the most highly expressed (Fig. 5).

Genome annotation did not reveal pathways for dissimilatory sulfate reduction nor sulfur oxidation. However, genes coding for assimilatory sulfate reduction, including ATP sulfurylase, adenylyl-sulfate kinase, 3´-phosphoadenosine-5´-phosphosulfate reductase, and sulfite reductase, were identified and those encoded by DWH *Bermanella* and DWH O. Desum v2 were the most abundant and most highly expressed (Fig. 5). Additionally, all non-DWH genomes and three DWH MAGs coded for assimilatory sulfate reduction, but these genes and transcripts were not abundant (Fig. 5). DWH *Bermanella* transport genes were the most abundant of any analyzed here, with MG RPKM values up to 981 and MT RPKM values of 163 (Fig. 5).

Phosphate cycling genes include alkaline phosphatase (APase), utilized in organophosphate acquisition, and phosphoenolpyruvate (PEP) phosphomutase, which is involved in phosphonate metabolism, were identified. APase was encoded by members of most clades, with DWH *Bermanella* genes being the most abundant and representing the only active transcription of these genes (Fig. 5). PEP phosphomutase was also encoded by all clades, except SAR324 (Fig. 5). No other phosphonate metabolism genes were annotated in any genome (Fig. 5). All clades had members that encoded phosphate transport genes (ABC type phosphate/phosphonate transport system) with DWH *Bermanella* encoding the most abundant and the only transcriptionally active transport genes (Fig. 5).

Siderophore production and transport genes were encoded by 29 genomes in all clades, except SAR324. DWH *Colwellia* siderophore-associated genes were the most abundant, recruiting the most MG and MT reads of any microbe in this study (Fig. 5). Additionally, iron acquisition and transport genes (including ferrous and ferric iron transport systems) were encoded by members of all clades (Fig. 5). These genes were most abundant in the proximal and distal samples with lower abundances in the uncontaminated station (Fig. 5).

### Chemotaxis and motility

Nearly all *Bermanella*, *Colwellia*, and *Cycloclasticus* encoded and expressed methylase, signaling, and receiver domains of methyl-accepting chemotaxis proteins (MCPs) and flagellar-associated genes, indicating the capacity for motility (Fig. 5). However, no UBA11654, and only one SAR324, encoded these genes. DWH *Bermanella* chemotaxis genes were the most abundant in this study, with maximum RPKM abundances of 394 and 321 from the distal and proximal station samples, respectively, while motility genes from DWH *Cycloclasticus* were the most abundant [684 and 413 in the distal and proximal sites, respectively; (Fig. 5)]. Fewer MG reads were recruited to chemotactic and motility genes in the uncontaminated sample compared to plume samples. Chemotaxis and motility genes were actively transcribed in plume samples (Fig. 5).

### Cold tolerance

Cold-shock genes in the CspA family were encoded by members of all clades (Fig. 5). The cold-shock genes of DWH *Bermanella* MAGs were the most abundant of the genomes analyzed here, particularly in the distal and proximal samples (Fig. 5). These same MAGs also had the highest levels of CspA transcription in this study with an RPKM >890 in the proximal sample and RPKM values <26 in the distal sample (Fig. 5). The remaining MAGs recruited fewer MG or MT reads (Fig. 5).

### The pangenome and clade core functions

Given the paucity of data available on the genomic content of deep-sea planktonic microorganisms, particularly those involved in hydrocarbon degradation, a core genome, or pangenome, was identified for all 38 genomes and separately for DWH MAGs only. Analysis of all genomes, which included 100,262,742 nucleotides, revealed that the core functions identified in the pangenome included acyl-CoA dehydrogenase, acetyl-CoA acetyltransferase, and enoyl-CoA hydratase/isomerase (Table S1). Short-chain alcohol dehydrogenase, utilized in methane oxidation and other metabolic pathways, would have been included with the pangenome, but the COG and Pfam annotations for these genes in one DWH *Bermanella* (OV003_3) were not consistent (as shown in Fig. S2). The remaining core functions consisted mostly of housekeeping genes (Table S1). The core genes of just the DWH MAGs included those in the full pangenome core and additionally citrate synthase and aldehyde dehydrogenase, which can be associated with hydrocarbon degradation as well as other pathways (Table S1).

## DISCUSSION

The first hydrocarbon-degrading microbes were cultured and described more than a century ago. In fact, much of what we know about hydrocarbon-degrading microbes, particularly those in the marine environment, stems from cultivation-based studies of microbes such as *A. borkumensis*, an aliphatic hydrocarbon degrader that is ubiquitous in marine ecosystems and blooms quickly when hydrocarbons are input to the environment (65, 66). Other Gammaproteobacteria such as *Cycloclasticus* (19) and *Oleispira* (67) were also isolated from the marine environment, and, like *A. borkumensis*, were shown to be obligate hydrocarbon-degrading (hydrocarbonoclastic) bacteria. These early studies provided important linkages between hydrocarbon degradation capabilities and taxonomy. During the DWH oil spill, ‘omics analyses provided new insights into the degradation capabilities of marine microbes, revealing the dominant pathways that were actively expressed *in situ* in the deep-sea plume [e.g., references (6, 8)]. However, unlike cultivation-based studies, except for single-cell genomics analyses presented in Mason et al. (6, 18), ‘omics analyses did not link physiology and identity given the lack of assembled and binned genomes from these data. Using genome assembly, phylogeny, and read mapping, we attempted to bridge the gap and link function and identity of hydrocarbon degraders indigenous to the deep-sea in the GOM and by inclusion of non-DWH genomes, sampled outside the GOM, to extend findings to the global ocean.

Consistent with 16S rRNA-based studies, the Gammaprotobacteria that were reported as being abundant members of the plume microbial community, such as *Oceanospirillales*, *Colwellia,* and *Cycloclasticus* (6, 7, 9–11, 13), were represented in the MAGs presented here. We would note that the 16S RNA gene in DWH *Bermanella* MAG BM58_1, the most abundant microbe in our analyses, was 99.93% similar to DWH *Oceanospirillales* clone BM580104 (HM587889.1) and 99.33% similar to DWH *Oceanospirillales* clone OV01102/03 (HM587890.1) (9). This MAG was also 98.36% similar to the dominant *Oceanospirillales* in the plume 16S rRNA gene pyrotag data (6). Collectively, this suggested that the dominant players in the DWH oil spill were captured in the assembled MAGs and in the genome, gene, and transcript abundances that were observed, particularly in regard to *Bermanella*. In addition to the dominant players in the plume, we also assembled Gammaproteobacteria classified as UBA11654 and representatives of the SAR324 phylum, neither of which have been previously recognized as members of the plume community. While less abundant than the aforementioned clades, members of UBA11654 and SAR324 encode genes that were, in some cases, abundant and actively expressed.

DWH MAGs were most similar to other DWH MAGs within the same clades; however, there were several notable exceptions. For example, using a different assembly method than what we used, the DWH O. Desum v2 genome was highly similar to DWH MAGs (*Bermanella*), as were some MAGs described in Hu et al. (36), and, in the case of *Colwellia*, isolates such as *Colwellia psychrerythraea* 34H, which was cultured from Arctic sea ice (40). Additionally, DWH SAR324 MAGs were closely related to SAR324 from the South Atlantic at 800 m depth (67) and from a hydrothermal plume in the Atlantic (44) (Fig. 1). The relatedness, which in some cases was high, of these DWH MAGs to microbes sampled from a broad geographic distribution suggested that the genomic content encoded in the DWH MAGs may be more broadly representative of metabolic schemes employed by microbes in the global ocean, rather than just those residing in the deep-sea in the GOM. These additional genomes are medium to high quality (Table 1) which is particularly important if our MAGs were less than complete, as was the case with DWH *Colwellia*.

All 38 genomes analyzed encoded hydrocarbon degradation, despite the fact that the majority of the non-DWH plume microbes included in our analyses were sampled from non-hydrocarbon-contaminated environments that geographically spanned the global ocean and were inclusive of the surface to deeper in the water column, and marine sediments. The ability to degrade aliphatic and aromatic hydrocarbons was observed, with some pathways that appeared to be clade specific. For example, all *Bermanella* genomes coded for non-gaseous *n*-alkane degradation, all *Cycloclasticus* genomes coded for methane oxidation, and all SAR324 and all but one UBA11654 genomes coded for aromatic hydrocarbon degradation (Fig. 4). In contrast, long-chain *n*-alkanes and/or cyclic alkane degradation was a ubiquitous pathway encoded in members of all clades, except SAR324.

Our analyses revealed that methane oxidation was encoded in all *Cycloclasticus*, and in one *Colwellia*, which provided an important yet missing link between function and identity, particularly in regard to the DWH oil spill. For example, chemical (11, 14) and biological (6, 8) data suggested that methane oxidation was an important microbial metabolism, yet the identity of microorganisms carrying out this process remained enigmatic.

Methane oxidation is largely undocumented in *Cycloclasticus*. For example, despite its close phylogenetic relationship with canonical methanotrophs (*Methylobacter* and *Methylomonas*), *Cycloclasticus pugetii*, a well-known aromatic hydrocarbon-degrading (naphthalene, phenanthrene, anthracene, and toluene) bacteria isolated from marine sediments, is not able to use methane for carbon (19). Using SIP, Redmond and Valentine (7) revealed that indigenous deep-sea *Cycloclasticus* sampled in the GOM deep-sea plume are capable of degrading gases as shown by incorporation of ^13^C-labeled propane and ethane, but not methane. Similarly, genomic and transcriptomic analyses of *Cycloclasticus* symbionts of mussels and sponges that reside at deep-sea oil and gas seeps can use propane, ethane, and butane, but not methane (38). Although *Cycloclasticus* is not known to degrade methane, GTDB taxonomy, which is based on the alignment of single-copy marker proteins, previously led to the *Cycloclasticus* clade being reclassified as a fourth family in the *Methylococcales* order (68). Given that all *Cycloclasticus* encoded *pmoABC* genes, suggesting a methanotrophic lifestyle, our results support the reclassification to the *Methylococcales*, which are a clade of canonical methanotrophs. Additionally, protein tree analyses supported the close phylogenetic relationships between DWH MAGs presented herein and two MAGs (*Cycloclasticus* sp. symbiont of *Poecilosclerida* sp. and *Cycloclasticus* sp. symbiont of *Bathymodiolus heckerae* specimen P, see Fig. S1) from Rubin-Blum et al. (69). Taken together, the data suggested that, like other *Methylococcales*, the *Cycloclasticus* analyzed in this study appear to be capable of gaseous hydrocarbon degradation, including methane.

*Colwellia* that were capable of consuming gases were reported in Mason et al. (18) through SAG assembly and gene annotation and in Redmond and Valentine (7) who used SIP and 16S rRNA gene sequencing. In Mason et al. (18), the *Colwellia* SAG was hypothesized to have a butane monooxygenase, which is known to preferentially oxidize gases other than methane. Using SIP, Redmond and Valentine (7) reported that *Colwellia* was consuming ethane and propane, but not methane. Here, we found evidence that extends gaseous hydrocarbon degradation capabilities for *Colwellia* to include methane oxidation.

Non-gaseous *n*-alkane degradation was the most abundant hydrocarbon degradation pathway at the time we sampled, which is consistent with previous reports that suggested this was the most abundant metabolic strategy employed by indigenous microbes to degrade DWH oil in the deep-sea plume (6, 8). Degradation of medium-chain *n*-alkanes (C_5_–C_26_) was largely attributed to *Bermanella* that encoded the AlkB protein (6). While *alkB* was encoded in several genomes, none of the DWH *Bermanella* possessed this gene. Instead, the DWH *Bermanella* in the plume encoded and expressed CYP153. We also identified rubredoxin as present and expressed in all DWH *Bermanella* MAGs and two non-DWH *Bermanella*. In *A. borkumensis*, rubredoxin is part of the complete AlkB operon, and is the second step in alkane oxidation (62). Rubredoxins are integral electron transfer components necessary for alkane hydroxylation by *alkB* (61, 70). It has been suggested that it could theoretically serve the same function with CYP153 (61). The presence of CYP153 and rubredoxin genes in these genomes suggested that *n*-alkane degradation proceeds from CYP153 to rubredoxin, which would represent the two-domain architecture for *n*-alkane degradation (61), and is a likely pathway used by the dominant microbes to degrade oil in the deep-sea consortium.

Additionally, three DWH *Bermanella* MAGs that encoded CYP153 also encoded cytochrome P450, ferredoxin, and ferredoxin reductase, suggesting a three-domain CYP153 architecture for *n*-alkane degradation (61). Furthermore, if rubredoxin functions similarly when paired with CYP153 as it does with AlkB, this would indicate that DWH *Bermanella* used two pathways to degrade non-gaseous *n*-alkanes. The fact that DWH *Bermanella* encoded CYP153 rather than AlkB as originally proposed [e.g., reference (6)] may be due to the high abundance of C_8_–C_16_ *n*-alkanes compared to longer-chain *n*-alkanes in Macondo oil that has been reported [e.g., see Fig. 5 in reference (5)]. This hypothesis is supported by heterologous expression experiments with *A. dieselolei* AlkB, CYP153, and AlmA (discussed below) in Liu et al. (63). They showed that CYP153 was upregulated in the presence of C_8_–C_16_ *n*-alkanes, while AlkB responded to C_12_–C_26_. The more focused range of *n*-alkanes that led to upregulation of CYP153 compared to AlkB reported by Liu et al. (63) may suggest that CYP153 is an enzyme that is more fine-tuned to C_8_–C_16_ *n*-alkane degradation, which is consistent with the profile of Macondo oil. Thus, degradation of non-gaseous *n*-alkane hydrocarbons using multiple previously undescribed pathways by DWH *Bermanella* likely gave this microbe an advantage in that the dominant *n*-alkanes it degrades were the most abundant in Macondo oil.

Long-chain (>C_18_) *n*-alkane/cyclic degradation was a ubiquitous pathway encoded in all clades analyzed here, except SAR324. For example, the HAPMO/flavin-containing monooxygenase identified in DWH *Bermanella* MAGs herein was highly abundant and expressed in the plume samples. Maeng et al. (71) identified a flavin-containing *n*-alkane dioxygenase in *Acinetobacter* sp. strain M-1 which allowed for growth with medium- to long-chain *n*-alkanes C_10_ to C_30_.

Other flavin-containing monooxygenases, including AlmA, were annotated in DWH and non-DWH *Cycloclasticus* and UBA11654. Throne-Holst et al. (72) reported that AlmA in *Acinetobacter* sp. DSM 17874 supports its growth with long-chain alkanes up to C_36_. Liu et al. (62) carried out heterologous expression experiments and reported that AlmA in the marine hydrocarbon degrader, *A. dieselolei,* functions in long-chain *n*-alkane degradation. They further reported that AlmA was upregulated in the presence of long-chain *n*-alkanes ranging from C_22_ to C_36_. Putative *almA* genes have also been identified in *A. borkumensis* SK2 (73), which degrades long-chain *n*-alkanes up to C_32_. Furthermore, AlmA was recently identified in several *Oceanospirillales* MAGs that were assembled from the Mariana Trench (74). Although less abundant than medium-chain *n*-alkanes, long-chain *n*-alkanes up to C_38_ are constituents of Macondo oil (5). Thus, the high abundance of flavin-containing monooxygenases in DWH *Bermanella, Cycloclasticus,* and UBA11654 MAGs, with varying degrees of similarity with *almA* (30 to >60%), that were expressed in the plume, suggested that oxidation of long-chain *n*-alkanes up to C_36_ and perhaps longer was an important microbial degradation strategy used by indigenous microbes during the DWH oil spill. Furthermore, non-DWH *Bermanella, Cycloclasticus,* and UBA11654 MAGs from microbes sampled globally also encoded flavin-containing monooxygenases, including *almA*, suggesting long-chain *n*-alkane degradation is a ubiquitous pathway in hydrocarbon-degrading microbes in the global ocean.

Other hydrocarbon degradation pathways, such as consumption of cyclic alkanes and aromatics, were encoded in DWH and non-DWH genomes, with some clade-specific degradation strategies observed. For example, all members of SAR324 coded for aromatic hydrocarbon degradation, but no other hydrocarbon degradation pathway was observed in this group. Although genes coding for aromatic hydrocarbon degradation recruited metagenomic reads, active aromatic hydrocarbon-degrading microbes were not observed in the DWH plume. Yet, the ability to degrade these hydrocarbons appears to be global, with aromatic degradation pathways identified in samples from other parts of the GOM, the North Atlantic, the Red Sea, the North Sea, and from sediments and crustal fluids.

Beyond hydrocarbon substrate availability, the bioavailability of nutrients, including nitrogen and phosphorus, is a key factor in the speed and success of hydrocarbon degradation; therefore, the abundance and expression of nutrient acquisition and transport genes can also provide insight into the microbial hydrocarbon degradation process (1). Although there was no significant oxygen depletion inside or outside the plume, nitrate concentrations were significantly lower in the plume (9). Complete denitrification by *Colwellia* identified herein is one possible explanation for the difference in nitrate concentrations inside and outside of the plume. In addition to nitrate, DWH *Bermanella* encoded and expressed genes involved in sulfur and phosphate/phosphonate transport, suggesting mechanisms to obtain nutrients by the dominant *Bermanella*. Last, iron depletion in the plume was reported (75) and may have been alleviated through siderophore production by non-DWH *Bermanella* and *Colwellia,* and to a lesser extent in *Cycloclasticus*, or by siderophore scavenging by *Colwellia* (18, 40).

The high abundance of all *Bermanella, Colwellia*, and *Cycloclasticus* was likely due to many factors, including the ability to sense and move along a gradient. Specifically, the aforementioned clade members encoded MCP genes, which were abundant and highly expressed. Some MCPs have been found to aid in sensing alkanes and other substrates and therefore may be linked with hydrocarbon degradation (6, 74, 76–78). Furthermore, all clades encoded flagellar biosynthesis and motor genes involved in motility. *Cycloclasticus* motility genes were some of the most abundant and expressed across all samples. This clade has long been known to contain motile members (19, 20), which could enable movement towards degradable hydrocarbon substrates.

All clades encoded cold-shock proteins (Csps), such as CspA, which was one of the most highly expressed genes in our study. Csps are expressed in response to rapid temperature decrease (cold shock), as well as to a wider range of stressors including those related to pH, starvation, and oxidative tolerance (79). During stress, but specifically cold shock, cell membrane fluidity and enzyme activity decrease, leading to reduced efficiency of transcription and translation due to formation of nucleic acid secondary structures (79). Csps act as nucleic acid chaperones that may prevent these secondary structures from forming at these low temperatures or other less than optimal conditions (79). Thus, high levels of CspA gene expression indicated that plume microbes were attempting to counteract the decrease in efficiency in transcription and translation, facilitating continued hydrocarbon degradation in the low-temperature deep-sea plume and may generally be indicative of a strategy widely used by marine microbes that reside in low-temperature environments.

The DWH pangenome, or core genes, included genes and pathways that are involved in hydrocarbon degradation. For example, aldehyde dehydrogenase can facilitate steps in gaseous and non-gaseous *n*-alkane oxidation and conversion of fatty aldehydes to fatty acids for incorporation into the beta-oxidation pathway (8, 73). Oxidation of alkanes results in formation of fatty acids which are degraded in the beta-oxidation pathway (73). Additionally, citrate synthase is involved in the tricarboxylic/glyoxylate pathway, which can be a final step of aromatic hydrocarbon degradation or can take place after fatty acid beta-oxidation (8). Furthermore, the pangenome included multiple steps of the beta-oxidation pathway which is the primary metabolism pathway for fatty acids derived from degradation of alkanes and aromatics (8). Rivers et al. (8) showed that beta-oxidation was one of the main pathways in which alkanes and aromatics were metabolized. Here, these transcripts were either enriched or significantly enriched as were transcripts of genes involved in the initial hydrocarbon oxidation steps. These core pangenome genes suggested that marine microbial hydrocarbon degradation is broadly distributed across multiple microbial clades. Linking cross-clade hydrocarbon degradation core genes with clade-specific hydrocarbon degradation genes/pathways discussed above provided missing information that identifies both the microbe and pathway used in degrading specific hydrocarbon substrates. These findings help resolve previous studies that linked hydrocarbon degradation to the dominance and succession of specific microbial groups over the course of the DWH oil spill (7, 9–12). Moreover, the inclusion of non-DWH genomes in this study expands the novel insights on the identity and function of uncultured marine hydrocarbon-degrading microbes to the global ocean.

## Data Availability

Metagenomic reads are publicly available in NCBI (PRJNA336903-05), in IMG Gold Study ID Gs0063184, and on the Mason server http://mason.eoas.fsu.edu in the DWH_plume directory. Amplified cDNA reads are publicly available in NCBI (PRJNA839076) and on the Mason server in DWH_plume. MAGs assembled and analyzed herein are available in NCBI under sample BM58 (PRJNA336904) MAG accession numbers JAVTJO000000000-JAVTJR000000000, sample OV011 (PRJNA336903) MAG accession numbers JAVTJS000000000-JAVTJW000000000, sample OV003 (PRJNA336905) MAG accession numbers JAVTJX000000000-JAVTJZ000000000, and on the Mason server in DWH_plume.

## SUPPLEMENTAL MATERIAL

Fig. S1 and S2 aem.01224-24-s0001.pdf Bacterial single-copy marker protein phylogenetic tree, and heatmap of abundance and expression of genes involved in methane oxidation.

Tables S1 and S2 aem.01224-24-s0002.xlsx Pangenome analyses.

ASM does not own the copyrights to Supplemental Material that may be linked to, or accessed through, an article. The authors have granted ASM a non-exclusive, world-wide license to publish the Supplemental Material files. Please contact the corresponding author directly for reuse.
