# Supplementary material for: Novel, active, and uncultured hydrocarbon-degrading microbes in the ocean
Source: Appl Environ Microbiol. 2024 Aug 23;90(9):e01224-24. doi: 10.1128/aem.01224-24 (PMC11409719; doi:10.1128/aem.01224-24)
Supplement: Fig. S1 and S2 — Bacterial single-copy marker protein phylogenetic tree, and heatmap of abundance and expression of genes involved in methane oxidation. [file aem.01224-24-s0001.pdf]

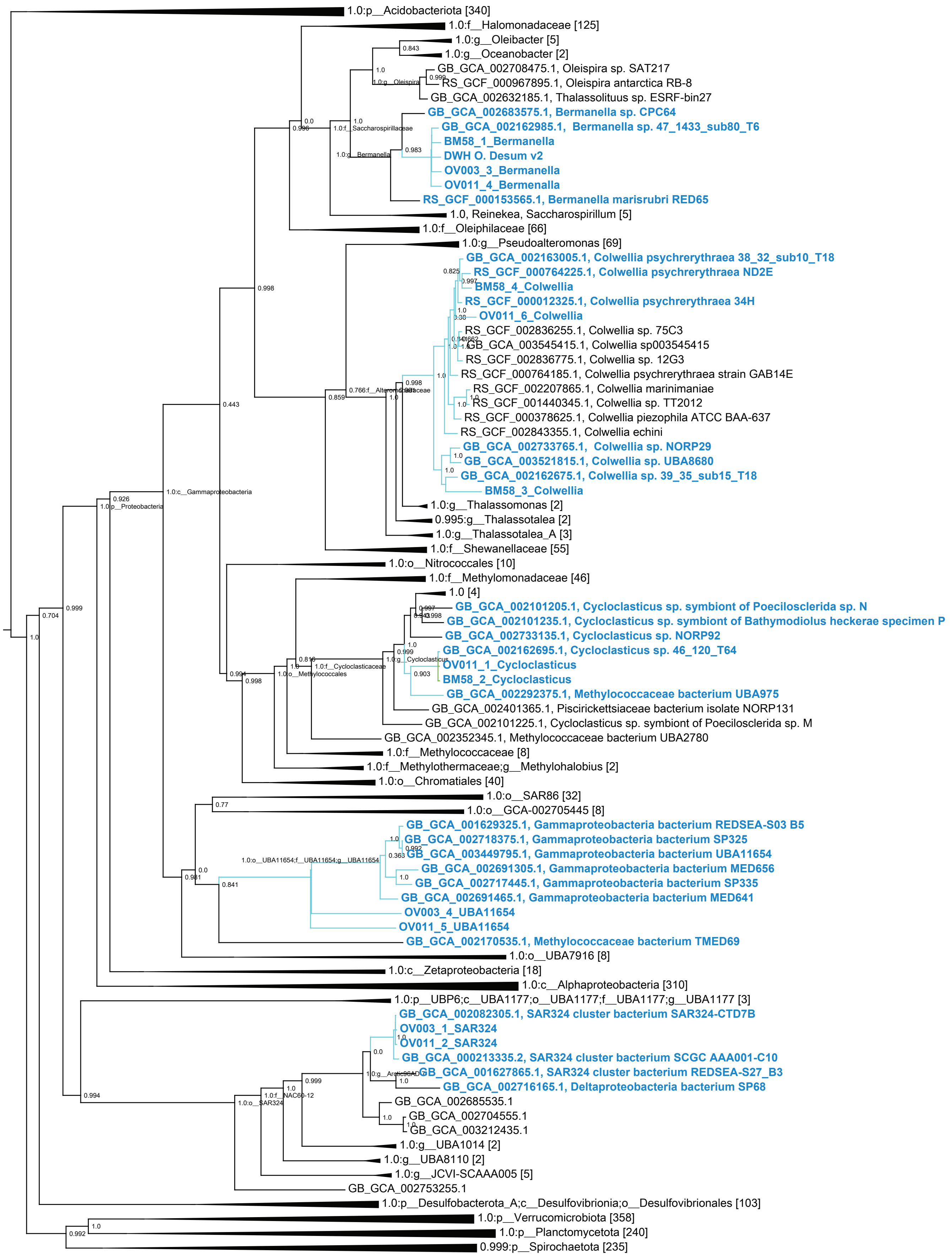

Supp. Fig 1. Bacterial single-copy marker protein phylogenetic tree from GTDB showing only relevant microbes, not all nodes in the original tree. Blue text denoting DWH MAGs and their close relatives

.

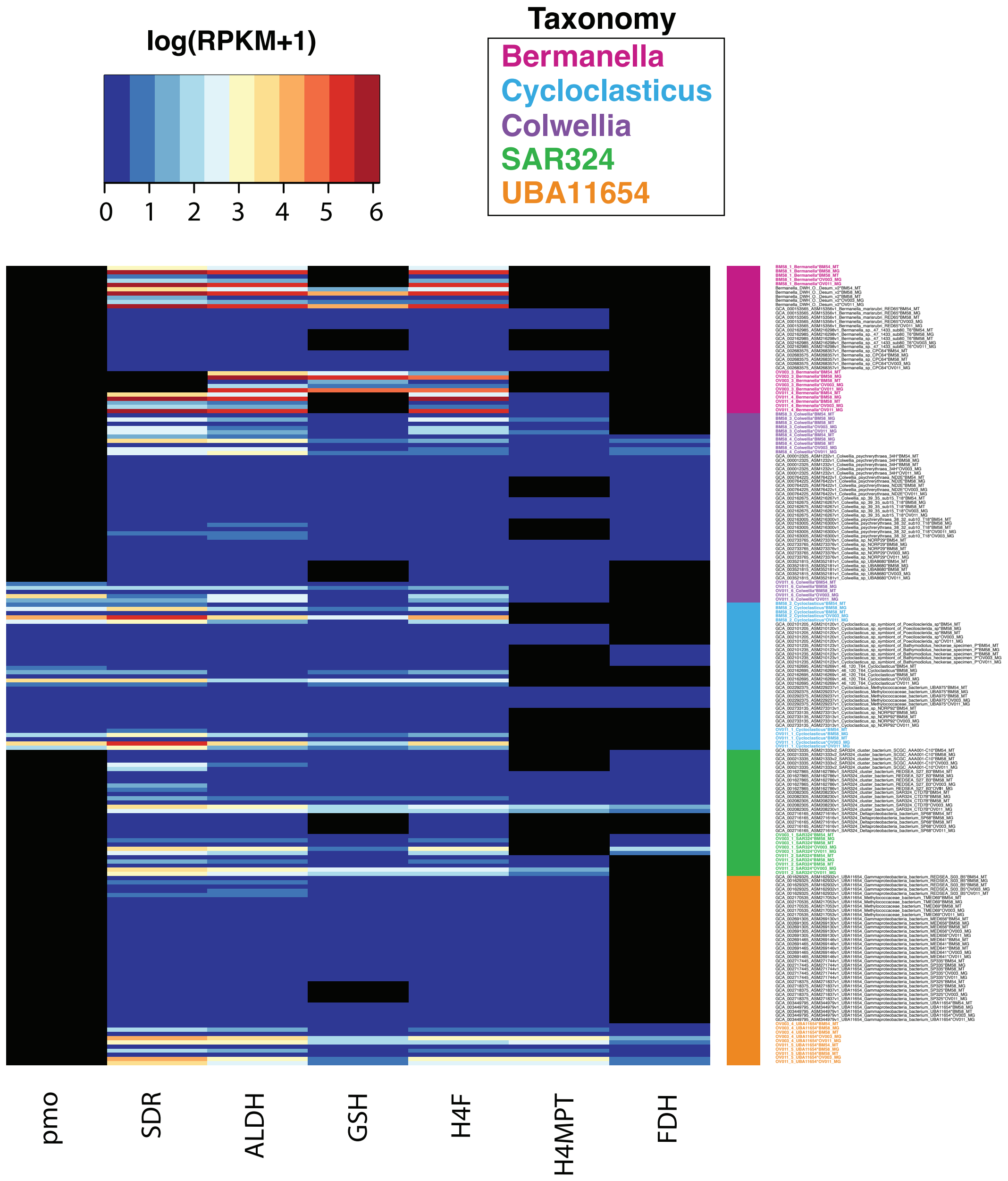

Supp. Fig 2. Heatmap of abundance and expression of genes involved in methane oxidation from read recruitment. Enzyme abbreviations are: methane monooxygenase (pmo), short-chain alcohol dehydrogenase (SDR), aldehyde dehydrogenase (ALDH), glutathione-dependent pathway (GSH), tetrahydrofolate pathway (H4F), tetrahydromethanopterin pathway (H4MPT), formaldehyde dehydrogenase (FDH). DWH MAG genome names are shown in bold text and color coded by taxonomy. Black cells indicate that the corresponding gene was not encoded in that genome.
